# Supplementary material for: Endophytic Colonization of Beauveria bassiana Enhances Drought Stress Tolerance in Tomato via “Water Spender” Pathway
Source: Int J Mol Sci. 2024 Nov 7;25(22):11949. doi: 10.3390/ijms252211949 (PMC11594164; doi:10.3390/ijms252211949)
Supplement: Supplementary file 1 [file ijms-25-11949-s001.zip › ijms-3285278-supplementary.pdf]

Supplementary Materials:

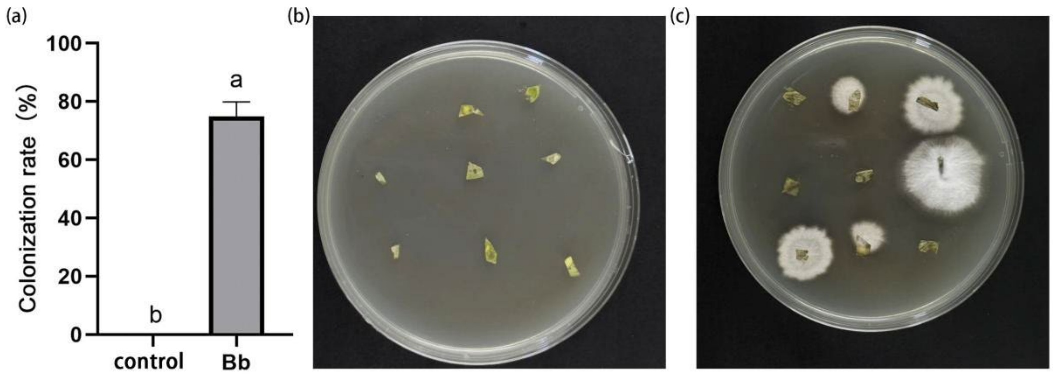

Figure S1. Detection of *B. bassiana* colonization in tomato leaves. (a) the rate of colonization in the tomato leaves. (b) PDA plate detection of *B. bassiana* in control tomato leaves not treated with *B. bassiana*. (c) PDA plate detection in tomato leaves treated with *B. bassiana*. Means represent the average of all replications.

Table S1. Primer sequences used to examine expression of genes related to stomatal development and drought stress tolerance pathways.

Gene ID	Primer sequence（5′to 3′）
Actin 7（ACT）	F：GGTATCCACGAGACTACCTACA
	R:TGCTCATACGGTCAGCAATAC
Solyc08g061560.2	F:CTGCTAAAACCCCACAACCC
	R:ACCTCACTAACTCAGCTCCT
Solyc12g042760.1	F:GTGCTCTAGTCGATGCTCCA
	R:GTTCCCCGTAGCCCTCATAA
Solyc09g091760.1	F:TGAAGAGTAGTGAGGAAGTTGAG
	R:TGAACTTGGCATGAGAGACCT
AOS	F: CAGGCTTCGGTGTCTGAGA
	R:GACTTTTTGGGCTGGGAGTT
APX2	F:AGTGTTATCCGACGGTGAGC
	R: TCTCAGCATAATCGGAGCAC
NIR1	F:GTGGGCAAGCAATAATCGAG
	R:CTGTCCAGTGCATCCTCACT
PSY	F:CCCGTCGACTACGAAAAAGA
	R:GTTTCTCATGCAAGGCGTAG
FAB2	F:GGGGATGASTTTCTGGTGAGA
	R:ACACACTAGGCAAGGGAAAAA

[1] Annealing temperature was set as 95 °C.
